# Will SARS-CoV-2 Infection Elicit Long-Lasting Protective or Sterilising Immunity? Implications for Vaccine Strategies (2020)

**DOI:** 10.3389/fimmu.2020.571481

**Published:** 2020-12-09

**Authors:** David S. Kim, Sarah Rowland-Jones, Ester Gea-Mallorquí

**Affiliations:** ^1^ Medical Sciences Division, University of Oxford, Oxford, United Kingdom; ^2^ Viral Immunology Unit, Nuffield Department of Medicine, University of Oxford, Oxford, United Kingdom

**Keywords:** SARS-CoV-2, COVID-19, protective, immunity, vaccine, immunogenicity

## Abstract

In December 2019, an outbreak of a novel coronavirus (SARS-CoV-2) in Wuhan, China resulted in the current COVID-19 global pandemic. The human immune system has not previously encountered this virus, raising the important question as to whether or not protective immunity is generated by infection. Growing evidence suggests that protective immunity can indeed be acquired post-infection—although a handful of reinfection cases have been reported. However, it is still unknown whether the immune response to SARS-CoV-2 leads to some degree of long-lasting protection against the disease or the infection. This review draws insights from previous knowledge regarding the nature and longevity of immunity to the related virus, SARS-CoV, to fill the gaps in our understanding of the immune response to SARS-CoV-2. Deciphering the immunological characteristics that give rise to protective immunity against SARS-CoV-2 is critical to guiding vaccine development and also predicting the course of the pandemic. Here we discuss the recent evidence that characterises the adaptive immune response against SARS-CoV-2 and its potential implications for the generation of memory responses and long-term protection.

## Introduction

Severe Acute Respiratory Syndrome Coronavirus 2 (SARS-CoV-2) is a novel coronavirus that appeared in Wuhan at the end of 2019 and rapidly escalated into the global pandemic of COVID-19, the disease that results from infection. Similar to previous recently-emerged coronaviruses, SARS and Middle East Respiratory Syndrome (MERS), SARS-CoV-2 is likely to have originated from a zoonotic transmission from bats (1). A recently discovered bat-derived CoV, RmYN02, was found to share 93.3% whole genome identity with SARS-CoV-2 and 97.2% identity in *1ab*—the longest coding gene (1). Typically, bats are the natural reservoir for coronaviruses, although transmission to humans often occurs *via* an intermediate host, which is still under debate for SARS-CoV-2. The pangolin, an animal used in traditional Chinese medicine, has been proposed as intermediate host due to a strong similarity in the receptor binding domain (RBD) between SARS-CoV-2 and pangolin coronavirus (2).

Genome sequencing studies of SARS-CoV-2 showed high levels of whole genome conservation (>99%) across 739 sequences reported on GISAID (3), which suggests that major mutations may be detrimental for viral fitness. A comprehensive analysis of the mutations found in SARS-CoV-2 has been published by Li et al. (4). A single mutation, D614G, affecting the viral spike protein, emerged in Europe and became the dominant circulating virus: this variant has been reported to increase viral infectivity but not affect disease severity (5). SARS-CoV-2 also shares 79%–82% of its genome with SARS-CoV, which was responsible for the 2003 SARS outbreak and is the most closely related coronavirus known to infect humans (6).

SARS-CoV-2 is an enveloped virus with a 30 kb single positive stranded RNA genome (7). It contains 12 canonical open reading frames (ORFs) that are translated either from genomic or subgenomic RNAs by the host cell upon entry (8). Interestingly, recent high-resolution map of coding regions has identified 23 other ORFs. ORFs 2, 4, 5 and 9a encode structural proteins (8). These proteins are the spike (S), the envelope (E), the membrane (M), and the nucleocapsid (N). The rest of the genome encodes non-structural proteins (NSP), such as the RNA dependent RNA polymerase, protease, and helicase, as well as other ORFs that act as accessory proteins, the functions of which are less well understood but assist in the completion of the viral cycle. For example, the NSP1 protein enables immune evasion by supressing host gene expression (9) and ORF7a counteracts host restriction factor Bone Marrow Stromal Antigen 2 (BST2) (10), similar to what has been described for SARS-CoV (11).

Both SARS-CoV and SARS-CoV-2 target the same receptor to infect target cells, ACE2 (Angiotensin-converting enzyme 2), through the highly conserved RBD in the S protein (12, 13). The S protein is composed of two functionally distinct domains; subunit S1, containing the RBD, engages with the ACE2 host cell receptor and the S2 subunit mediates fusion between the viral and the host cell membrane (14, 15). For fusion to occur following ACE2 binding the S protein is cleaved by the TMPRSS2 protease between the S1 and S2 subunits, which triggers fusion into the cell (16). This furin-like cleavage site (FCS) is uniquely present in the S protein of SARS-CoV-2, which might contribute to the significantly greater infectivity of SARS-CoV-2 compared to other known beta-coronaviruses (17). Alternatively, the viral particle can be endocytosed and enter the endosome/lysosomal pathway, where cathepsin L has been found to activate S protein and trigger fusion (15). Other host factors have been suggested to facilitate SARS-CoV-2 cell entry (18–24). Interestingly, Neuropilin-1 (NRP1), highly present in human respiratory and olfactory epithelium, has been shown to potentiate SARS-CoV-2 infectivity in the presence of ACE2 and TMPRSS2 by interacting with the furin-cleaved spike (25).

SARS-CoV-2 has been shown to bind ACE2 with 10-20 fold higher affinity than SARS-CoV, which may explain its greater transmissibility (14). The aforementioned D614G mutation in Spike appears to increase the proportion of Spike trimer components in the “open” conformation, which facilitates ACE2 binding and potentially confers greater infectivity (26). The S viral protein is the main focus for vaccine design as it is known to be highly immunogenic, particularly the RBD region. Moreover, the S protein is indispensable for viral entry and therefore its targeting may reduce or prevent infection. For SARS-CoV, vaccination with the S protein alone was shown to induce an immune response that was likely to be protective (27, 28): for this reason most current vaccine strategies against SARS-CoV-2 are based on the immunogenicity of S. Other structural viral proteins, like the nucleocapsid, have also generated interest, as they were studied in SARS-CoV infection and showed some immunogenicity (29).

The majority of individuals infected with SARS-CoV-2 experience mild-to-moderate disease. Although exact numbers are still debated, a systematic review analysis of 79 published studies found asymptomatic cases to be 20% of PCR-confirmed cases (30). Severe cases, defined as those requiring hospitalisation, are estimated to be around 14% of all confirmed cases. COVID-19 symptoms are diverse, but common manifestations include fever, dry cough, fatigue, loss of taste and/or smell, diarrhoea, and breathlessness (31). Age, sex, and underlying comorbidities, such as diabetes and hypertension, have been associated with disease severity (32). Patients over 65 years old have an odds ratio of 3.4 for requiring hospitalisation compared to 18–44 year olds (33). Critical COVID-19 patients often undergo a sudden clinical deterioration 7–10 days after symptom onset and present with features of acute respiratory distress syndrome (ARDS), along with lymphopaenia and an elevation of inflammatory markers (34, 35). It is important to note that some patients continue to suffer from symptoms for months after the infection has resolved.

The reasons behind the different outcomes are unclear. The main hypothesis is that a dysregulated immune response, probably at the early stages of infection, leads to systemic hyperinflammation (cytokine storm) that may be driving the ARDS and multi-organ damage observed in severe disease (34, 35). However, the reasons why there is loss of control of what is usually a tightly-regulated inflammatory response are still being investigated. Inborn defects in the Type I Interferon (IFN) pathway and anti-IFN auto antibodies have been found in life-threatening COVID-19 (36, 37).

Differences in the viral load of the initial inoculum, along with differences in the genetic sequence of the founder virus, have been proposed as potential factors that may influence overall patient outcome, given that a higher viral load at infection may trigger a stronger immune response. So far, the D614G mutant, associated with higher viral loads and greater viral dissemination, has not been found to correlate with disease severity (5).

A key question yet to be addressed is whether SARS-CoV-2 infection induces long-lasting protective immunity, and if so whether it will simply protect from severe disease or provide sterilising immunity. Our understanding of the immune correlates of protection for SARS-CoV-2 and their durability is limited to very recent data and depends mainly on knowledge gained from SARS-CoV, the most closely related virus known to affect humans. Long-lasting memory T cells to the genetically similar SARS-CoV have been revealed to be still detectable in convalescent patients 17 years after the SARS epidemic (38).

To date, few large-scale studies have characterised the immune response in recovered SARS-CoV-2 patients and it is too soon to evaluate the longevity of the response and its characteristics. The first challenge studies in macaques showed that SARS-CoV-2 infection may result in the development of protective immunity, when the animals were challenged soon after resolution of the primary infection (39, 40). However, a number of cases of reinfection in humans have now been reported a few months after initial infection, challenging the idea of long-lasting protective immunity (41–47).

In this review, we explore the current evidence for long-lasting immunity in SARS-CoV and the immunological parallels with SARS-CoV-2. We also examine the current literature characterising the immune response to SARS-CoV-2 and discuss its potential implications for current and future vaccine strategies. We aim to propose a perspective on whether protective immunity is likely to develop in individuals who have recovered from COVID-19 infection and for how long we might expect it to last. Some of the papers we have included were available on pre-print servers at the time of publication and therefore have not yet been peer-reviewed.

## Humoral Immune Response

### Antibodies Induced in SARS-CoV and SARS-CoV-2 Infection

**Figure 1:**
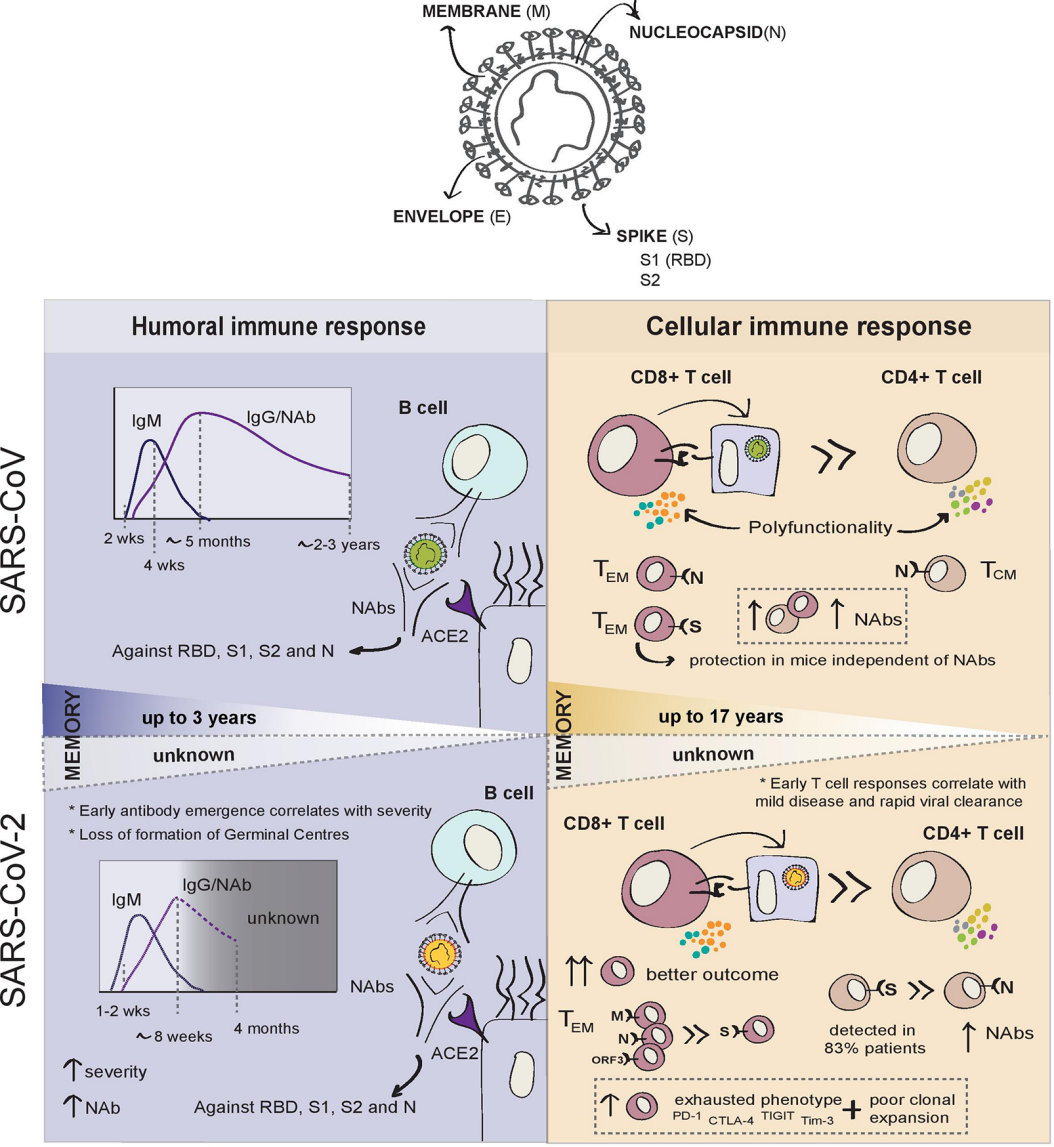
Summary of adaptive immune responses to SARS-CoV and SARS-CoV-2.

In the previous SARS epidemic, seroconversion was documented at 2 weeks post-onset of symptoms in the majority of patients with SARS-CoV infection (**Figure 1**). IgM antibodies appeared during acute infection, then, with progressive class-switching to IgG, IgM, and IgG titres increased in parallel during the first weeks after infection. Coinciding with the resolution of infection, IgM titres were shown to wane gradually 4 weeks after symptom onset. IgG levels peaked 4 months later, and both neutralising and non-neutralising antibody titres were shown to remain detectable for at least 2 years. Non-neutralising IgG antibodies became undetectable after 24 months in a small proportion of patients (48).

Recovered SARS patients were found to have high titres of neutralising antibodies (NAbs) directed against the receptor binding domain (RBD) of the S protein (defined as residues 318–510). These specific antibodies maintained high titres up to 5 months post-infection and remained measurable in sera at 24 months follow-up, although notably decreased after 16 months (48).

The immune correlates of the humoral response in SARS-CoV are likely to provide a good basis on which to predict antibody responses for SARS-CoV-2, especially given that the S protein is highly conserved between the two viruses (76% amino acid sequence homology) and antibodies targeting the RBD region appear to be crucial for neutralisation (15). S-based neutralising antibodies generated in humans against SARS-CoV-2 are likely to be similar in structure and function, and to follow homologous kinetics to those produced in response to SARS-CoV.

To date, similarly to SARS-CoV, people infected with SARS-CoV-2 are reported usually to develop IgA and IgM antibodies 7–14 days after symptoms develop and, together with later IgGs, antibodies are present before the resolution of infection (**Figure 1**) (49). However, COVID-19 seroconversion can occur as early as 2 days after symptom onset (50). In one of the largest studies conducted 44%–56% of patients had developed IgG on day 7 after the onset of symptoms and IgG antibodies were present in 95% of patients by day 20 (51). More recent studies have observed a small decrease in IgG titres 8 weeks after symptom onset (49, 52), despite maintenance of high titres of antibodies against the Spike protein (53, 54). The longevity of the antibody response may depend on their targets. N and S specific IgG antibody titres were found to remain stable for at least 4 months post-diagnosis, while anti-N IgM increased markedly early in infection, before declining to undetectable levels at 2 months (55). For now, only one report has revealed the dynamics of the antibody responses 6 months after convalescence and found anti-S1 IgG and IgA to decrease over time, while anti-N antibodies remained stable. Interestingly, higher anti-N antibody titres correlated with the prevalence of post-infection symptoms (56).

### Neutralising Antibodies and Cross-Neutralisation

Neutralising antibodies are key to provide sterilising immunity against a second infection, in particular those targeting the S-ACE2 binding to prevent infection. Many *in vitro* and *in vivo* studies have shown that NAbs appear to control SARS-CoV-2 infection (57–59).

In SARS-CoV-2, NAbs have been reported from day 10–15 after symptom onset and correlate with S-targeting antibodies (54) with 90% of seroconverters having detectable NAbs up to 5 months after infection (55). There is a clear association between antibody titres, and NAbs levels, with disease severity (60). NAb titres show an inverse correlation with the patient’s lymphocyte counts, where low lymphocyte counts constitute a strong indicator of poor prognosis (61). Robust antibody responses have been found in mild-to-moderate COVID-19 (55), but others could not detect NAbs in 30% of patients (54), suggesting that, for some patients with mild or asymptomatic infection, NAb titres are either very low or non-existent. The titres of NAbs therefore appear to correlate directly with disease severity, but paradoxically, may provide higher protection against infection on subsequent exposure to SARS-CoV-2.

Most antibody responses in COVID-19 patients appear to target the S viral protein, in particular the S1 subunit and RBD region, which are thought to elicit the most potent neutralising effect (62). Isolated potent NAbs against the RBD were shown to reduce viral burden and to protect against infection in mice and macaques (63, 64).

Given that the RBD sequence is highly conserved between the two viruses, we would expect SARS-CoV-2 NAbs directed against RBD to persist for a similar length of time to that observed for SARS-CoV. Structures of S-specific antibodies bound to the SARS-CoV-2 spike reveal common epitopes and conserved features (65). Murine SARS-CoV S antibodies can inhibit SARS-CoV-2 cell entry, consistent with the similarities of the S protein between the two viruses (66). Serum from SARS-CoV recovered individuals during the 2003 outbreak has been shown to cross-react with the S ectodomain, S, RBD and S2 proteins of SARS-CoV-2 and neutralise SARS-CoV-2 *in vitro* (67, 68). More recently, RBD-targeting antibodies have been isolated in convalescent SARS-CoV plasma and were shown to cross-neutralise SARS-CoV-2 by targeting multiple conserved epitopes on the S protein (69). However, other reports in humans suggest that convalescent sera from SARS-CoV and SARS-CoV-2 recovered patients show limited cross-neutralisation between the two viruses. These differences were ascribed to seven critical residues that differ in SARS-CoV and SARS-CoV-2 S RBDs, despite 76% amino acid identity (15).

Despite the general capacity of SARS-CoV and SARS-CoV-2 antibodies to cross-react, their ability for cross-neutralisation may be very specific. Such specificity was demonstrated in plasma from subjects with either SARS-CoV and SARS-CoV-2 that contained antibodies able to cross-react in their binding of spike protein but not to cross-neutralise (70). The isolation of SARS-CoV-2 RBD NAbs showed that their neutralising capacity is directly correlated with the ability to compete with ACE2 for RBD binding, but they were unable to neutralise SARS or MERS RBD, despite showing cross-reactivity against their S proteins trimers (71). Moreover, antibodies against seasonal coronaviruses (HCoV) from pre-pandemic sera, showed no cross-neutralising activity for SARS-CoV-2 (72, 73).

The S2 domain of SARS-CoV-2 also appears to be highly conserved (88% sequence homology with SARS-CoV). Similarly to S1, antibodies generated against S2 in SARS-CoV infection were found to be cross-reactive to S2 in SARS-CoV-2 but none were neutralising (69).

COVID-19 patients produce a spectrum of antibodies to both structural and non-structural proteins of SARS-CoV-2. These antibodies, targeting NSP1, ORF3b, ORF7a, and ORF8, may mediate immune functions other than neutralisation that may be beneficial or harmful to the patient (74). Whether antibodies to non-structural proteins add to protection is not yet known.

## Humoral Protection

In humans, plasma memory B cells and monoclonal NAbs have been isolated from SARS-CoV-2 convalescent individuals (63, 71). Memory B cells may persist in plasma in the absence of detectable antibody titres and can then be recalled in the event of subsequent infection and confer protection (69). Based on the findings from SARS-CoV studies, memory B cells and antibody-mediated protection may last for up to 3 years, although the efficacy of such protection will likely fall with time as circulating antibody titres decay (69). Many trials have highlighted the potential protective effect of antibodies generated naturally during the course of SARS-CoV-2 infection by using convalescent plasma (CP) from recovered COVID-19 patients to treat critical cases (75–77). However, a recent multicentre, randomised and controlled trial found no difference in mortality or disease progression for those treated with CP (78). Although the efficacy of convalescent plasma is still under debate, this could suggest that the contribution of the humoral immune response may be limited for patients who are already severely ill.

Evidence that SARS-CoV-2 infection can induce protective immunity was first shown by Chandrashekar et al. in rhesus macaques studied soon after initial infection (39). Nine macaques infected with SARS-CoV-2 were re-challenged 35 days after primary infection had been cleared and showed significantly lower viral RNA measured in bronchoalveolar lavage (BAL) compared to challenge-naïve animals. This was accompanied by the effective induction of neutralising antibodies and cell-mediated virus-specific responses (39). The demonstration of protective immunity developing after SARS-CoV-2 infection in macaques gives reason to postulate that protective immunity can also be generated in humans following recovery or vaccination. However, the longevity of such protection is yet to be evaluated.

To date, among the few reinfection cases reported, one study found the humoral immune response to be defective during the second infection, suggesting that a failure of the humoral response during the first infection may account for susceptibility to reinfection (42). In addition, Chen et al. demonstrate a positive correlation between the magnitude of NAb titres and disease severity but also report immense heterogeneity between the NAbs generated—with 80.7% patients producing S1 specific NAbs and only 40% patients producing NAbs to both S1 and S2. Notably, asymptomatic patients failed to generate adequate titres of NAbs, and sera from these patients were unable to neutralise SARS-CoV-2 pseudovirions *in vitro* (79).

A matter of concern is that, in fatal COVID-19 cases, SARS-CoV-2 appears to impair the generation of germinal centres (GC), where, with the help of T Follicular Helper (T_FH_) cells, B cells can differentiate into memory B cells or long-lived plasma cells (80). Interestingly, extrafollicular B cell activation has been detected in severely ill patients and correlated with early antibody responses, multi-organ failure and death (81). Lack of GC formation during acute SARS-CoV-2 infection would seriously undermine the generation of long-lived antibody responses. It has been proposed that GC are blocked from forming due to excessive Tumour Necrosis Factor alpha (TNF-α) production in severe disease. Therefore, it is possible that the formation of a memory compartment is compromised for humoral responses because of an excessive inflammatory response.

It is important to understand the correlates of humoral protection and to consider the immunological implications for those individuals with undetectable NAb titres. T cell responses may potentially provide protection against subsequent infection in the absence of NAbs. Early studies appear to show that virus-specific T cell responses are robust and develop in asymptomatic or mild SARS-CoV-2 infections, even in the absence of an antibody response (82).

Evidence so far shows that humoral responses to both SARS and COVID-19 share a number of similarities. However, it will be crucial to characterise the SARS-CoV-2 humoral immune response in relation to disease severity and determine whether the kinetics and the breadth of the antibody response play a role in the resolution of primary infection and protection against reinfection. In COVID-19, the antibody response appears to be stronger in those with more severe symptoms, with higher NAbs titres correlating with disease severity (37), while NAb titres appear to be low or undetectable in young people and asymptomatic cases (62, 82), with 40% of asymptomatic patients becoming seronegative during convalescence (83, 84). This may suggest that protective immunity against COVID-19 is short-lasting in asymptomatic or mild cases, although NAbs have been detected irrespective of symptoms in a cohort of health care workers 4 months after infection (85). However, if protective immunity is short-lasting in asymptomatic cases, it may well be a reason for concern about any public health policies that rely on the generation of herd immunity following natural infection.

Data from SARS-CoV studies indicate that neutralising antibodies may be able to persist for up to 3 years (69). If the same holds true for SARS-CoV-2, regardless of whether the neutralising antibodies are naturally or vaccine-induced, this could provide a reasonable window of protection but not necessarily confer lifelong humoral immunity.

### Early Antibody Responses and Antibody-Dependent Enhancement

It has been a matter of debate as to whether certain pre-existing or early-generated antibodies may facilitate disease progression in both SARS and COVID-19 (86). The proposed mechanism is known as antibody-dependent enhancement (ADE), which enables the virus to gain cellular entry *via* Fc-receptor-mediated internalisation of non-neutralising antibodies bound to virus. ADE has been observed during infection with feline coronaviruses and a number of flaviviruses, which has raised concerns that it may also contribute to the immunopathology of both SARS and COVID-19 (87, 88). A number of animal studies during SARS-CoV vaccine development demonstrated the possibility of deleterious effects caused by ADE as a result of particular non-neutralising antibodies induced by vaccination (89, 90). However, these studies should be taken with caution, as complement and FcR-IgG engagement mechanisms are species-specific.

The first hypothesis for ADE is that pre-existing immunity to endemic strains of HCoV (HCoV-183 229E, HCoV-OC43, 184 HCoV-NL63, and HCoV-HKU1) that cause mild seasonal illness and, despite relatively limited homology to SARS viruses, may potentially share antigens with SARS-CoV-2 (53) and facilitate disease progression mediated by potentially cross-reactive antibodies (62). The rationale for antigenic cross-reactivity (or “original antigenic sin”) contributing to severe disease was based on the observation that a relatively small percentage of severe cases occur in children and young people. Given that fewer pathogenic HCoV are abundant, a previous infection with a closely-related endemic strain of coronavirus with similar immunological epitopes could be sufficient to trigger the production of cross-reactive antibodies in response to SARS-CoV-2 infection. If true, the induction of memory antibodies which fail to neutralise SARS-CoV-2 would not only fail to protect but could also facilitate viral invasion and worsen prognosis.

The second hypothesis for ADE is the early emergence of antibodies against SARS-CoV-2, as there is an increased mortality risk in patients with early emergence of IgG that could implicate a recall immune response (91). In one study, NAbs appeared to emerge early in a small proportion of SARS-CoV patients (17%), during the first 2 weeks of illness, but this group of early responders were shown to have an increased mortality rate (29.6%) (92). The authors suggested that the early emergence of neutralising antibodies correlated with the age of the patients and proposed that the early response implied priming effects from existing humoral memory against endemic strains of HCoV, which then led to higher mortality.

Antibody titres have been observed to correlate with disease severity for SARS-CoV-2, but some have suggested that this is likely to reflect a response to high viral loads rather than being the cause of pathology (62, 79, 93). However, a recent report has found not only an association between the rapid induction of humoral responses and disease severity, but also with an early N-biased antibody response in severe disease and a more balanced or S-dominant response in mild and moderate disease (94). This finding suggests that antibodies induced early against SARS-CoV-2 N could play a pathogenic role in the severity of COVID-19, so the ADE hypotheses should not be completely disregarded at this stage.

To our knowledge, recent results have provided the first direct evidence that anti-S IgG antibodies may correlate with disease severity in COVID-19 patients. Hoepel et al. showed *in vitro* that serum from severely ill patients formed complexes with SARS-CoV-2 Spike protein and triggered macrophages to release pro-inflammatory cytokines in an antibody-dependent manner. This induced long-lasting endothelial barrier disruption *in vitro*. The mechanism was found to be linked to an aberrant Fc glycosylation pattern, but the authors also showed that downstream Syk inhibitors could prevent macrophage activation (95).

The current evidence favours the argument that ADE is not the main driver in the pathological immune response against SARS-CoV-2, although it cannot be completely ruled out as a mechanism explaining disease severity in some patients. Arvin et al. have published a review where this is discussed in depth (96). Preliminary studies trialling treatment with convalescent plasma have so far shown no adverse effects in COVID-19 patients (75–77).

A recent pre-clinical study in a mouse model for SARS-CoV-2 failed to demonstrate ADE using recovered patient sera containing anti-SARS-CoV-2 RBD antibodies, although under the same conditions ADE was observed with Zika virus antibodies (97). In contrast to many of the small animal studies for SARS-CoV, no ADE was observed in the three rhesus macaque studies involving challenge with SARS-CoV-2 (39, 98, 99). These studies all demonstrate the induction of Th1 responses rather than Th2 responses following infection with SARS-CoV-2, immunisation with a S protein based viral vector (ChAdOx1) vaccine or with a DNA vaccine (39, 98–100). It is important to note that there are limitations to non-human primate models of ARDS and lung injury, due to differences between human and animal innate immune systems, risk factors, and co-morbidities (101).

To date, two case studies, in Nevada (United States) and another in the Netherlands, have reported patients with more severe clinical symptoms after SARS-CoV-2 reinfection—with one case leading to death (44, 46). This may re-ignite the debate about the role that ADE could play in poor patient outcomes. Strong humoral responses and B cell activity in severely ill patients may also suggest a pathogenic role for antibodies (102), but the use of convalescent plasma has so far not been shown to worsen disease outcome, with some evidence for a slightly better outcome in elderly patients (103). Based on current evidence, it is therefore unlikely that ADE is driving the severity of disease in SARS-CoV-2 infection. Nevertheless, the results from Hoepel et al. suggest that the possibility that some patients may develop a form of ADE should not be completely discounted as a mechanism for disease exacerbation in COVID-19, and therefore the outcomes of spike-based vaccines in human trials should be carefully evaluated.

### Cellular Immune Response

Cellular immune responses are critical for both the resolution of viral infections and the development of long-lasting immunity, which will usually last longer than humoral responses (**Figure 1**). Memory B cells can be recalled into antibody-producing plasmablasts in the event of infection. During the first SARS epidemic, both memory B cells and neutralising antibodies were found to persist in SARS-CoV patients up to 3 years post-infection (69). However, SARS-CoV-specific antibody levels in recovered patients were undetectable at 6 years post-infection while N-specific memory T cells could be detected at the same time point (104). Similarly, a later study showed that SARS-specific memory T cells persisted in blood at 11 years post-infection in three recovered patients (105). Recently, T cell responses to SARS have been detected 17 years after the epidemic and were shown to potently cross-react against SARS-CoV-2 (38). These data support the belief that memory T cells may critically contribute to long-term responses to SARS-CoV-2. However, it is not clear whether the level at which T-cell responses are maintained would be sufficient to provide protective immunity against reinfection, and this was not addressed in these studies.

Lymphopaenia is one of the defining clinical characteristics of severe COVID-19. During SARS-CoV-2 infection, both CD4^+^ and CD8^+^ T-cell depletion strongly correlate with the patient’s clinical course (38, 53, 106). The decrease of circulating T cells is probably due to recruitment into the lungs, as well as cell death. High numbers of infiltrating CD8^+^ T cells and macrophages have been found in the lung tissue in autopsies of patients with significant pulmonary damage (107). Interestingly, preferential depletion of Natural Killer (NK) cells and mucosal-associated innate T (MAIT) cells compared to other T cell subsets has been identified in severe COVID (108–111), also suggesting recruitment to sites of infection, and highlighting the potential role of unconventional T cells in disease pathogenesis. The breadth and magnitude of SARS-CoV-2 specific T cell response in convalescent patients has been correlated with disease severity (112). However in one acute infection study, the early appearance of T cells responses was linked with rapid viral clearance and less severe disease (94).

### SARS-CoV T Cell Responses

When assessing T cell responses to the whole SARS-CoV proteome in the previous SARS epidemic, the majority of immunogenic epitopes were found within (but not limited to) the structural proteins, particularly in the S and N proteins, with the S protein inducing the greatest response (113). Among 70% of T cell responses induced against all structural proteins, 41% of those were elicited by the S protein for SARS-CoV (104, 113). In the recent study reporting long-lasting T cell responses in former SARS-CoV patients, two specific regions of the N protein (1-215aa and 206-419aa) were shown to elicit T cell responses in the majority of the individuals studied. In this same report, other non-structural proteins from ORF1 (NSP7 and NSP13) were also shown to elicit a response, but not in the majority of individuals (38). The role of these long-lasting T cell responses as correlates of protection against re-infection is yet to be determined.

From the few studies that identified antigen-specific CD4^+^ T cells from SARS-CoV recovered patients, responses were generated predominantly against the S and N proteins (114, 115). Interestingly, while N -specific CD4^+^ T cells exhibited a central memory (CD45RA^-^ CCR7^+^ CD62L^-^), N-specific CD8^+^ T cells showed effector memory (CD45RA^+^ CCR7^-^ CD62L^-^) phenotypes (115, 116). SARS-CoV-specific CD8^+^ T cell responses were far greater in magnitude than CD4^+^ responses and both CD4^+^ and CD8^+^ exhibited a memory phenotype characterised by higher proportions of polyfunctional T cells (113). High levels of CD4^+^ T cell responses and S-specific NAbs correlated with disease severity, but total and CD8^+^ T cell responses did not correlate with clinical outcome (113).

### SARS-CoV-2 T Cell Responses

In SARS-CoV-2, depletion of both circulating CD4^+^ and CD8^+^ T-cells is strongly associated with the patient’s clinical course, but the magnitude of the virus-specific T cell response was found to be proportional to disease severity in convalescent patients (112). The level of T cell responses strongly correlates with NAb titres, consistent with a T-cell-dependent NAb response, whilst higher serum Th2 cytokines (IL-4, IL-5, and IL-10) correlated with mortality rate (117, 118). T cell responses during acute infection to SARS-CoV-2 waned at 1 month post-infection, as expected after the resolution of infection (94). Interestingly, a recent longitudinal analysis found that although the magnitude of T cell responses wanes over time, the number of convalescent individuals with detectable T cell responses had actually increased 6 months post-infection. The authors also found that those still having post-infection symptoms had the most pronounced decrease in T cell responses, reinforcing the idea that T cell responses are critical to the resolution of SARS-CoV-2 infection (56).

Severe COVID-19 correlates with preferential CD8^+^ lymphopaenia as compared to loss of CD4^+^ T-cells (119). The CD8^+^ T-cell response has been shown to be critical for resolving SARS-CoV-2 infection, although virus-specific CD4^+^ T-cells have been detected more often than specific CD8^+^ T-cells in recovered COVID-19 patients (38, 53, 106). The total CD8^+^ T cell count, including the numbers of effector memory CD8^+^ T cells, negatively correlates with stage of disease progression in COVID-19 patients (93, 120). The cytotoxic phenotype of SARS-CoV-2-specific T cells also appears to correlate with disease severity during acute infection, and virus-specific T cells from recovered COVID-19 individuals are polyfunctional and exhibit a stem-like phenotype (82).

There is some controversy about the phenotype of CD8^+^ T cells across the spectrum of disease, with some reports finding them exhausted with decreased polyfunctionality and cytotoxicity (121–123).

The severity of disease has been associated with a marked T cell exhaustion phenotype together with poor clonal expansion (102), while moderately-ill patients show robust T cell expansion and higher proportions of highly cytotoxic effector T cell subsets, as well as CD4+ and CD8+ T cells expressing granulysin and CD160^+^ Natural Killer T (NKT) cells during convalescence (102). Exhausted COVID-19 CD8^+^ T cells express elevated levels of PD-1, CTLA-4, TIGIT, and Tim-3 (122, 123), but Kusnadi et al. showed that the exhausted phenotype in memory CD8^+^ T cells was more pronounced in mild disease, as compared to severe COVID-19, where CD8^+^ T cells appeared to be more polyfunctional and exhibit a pro-survival signature (124). The authors suggest that the failure to establish an exhaustion imprint may lead to the dysregulation of the T cell response and greater disease pathogenesis, which may imply that a distinct phenotype of CTL response is required for durable protection (124). SARS-CoV-2-specific CD8+ T cells in recovered patients were found to predominantly be long-lived T_EMRA_ cells, characterised by the re-expression of the naïve marker CD45RA and biased towards the CD27+CD28+ subset. For this reason, it has been suggested that SARS-CoV-2 specific CD8+ T_EMRA_ cells are likely to contribute to long-lasting protective immunity.

However, when analysed using functional assays, antigen-specific CD8^+^ T cells presented similar polyfunctional profiles in both severe and mild cases (112). In general, most of the single-cell studies performed find an effector phenotype for CD8+ T cells, with effector memory and terminal effector CD8^+^ T cells showing increased clonal expansion in peripheral blood, along with upregulated inflammatory markers consistent with their antiviral activity (120).

Broader and stronger SARS-CoV-2-specific T cell responses were found in patients recovered from severe disease, when compared to those who experienced only mild symptoms. In patients with mild infection, the ratio of CD8^+^:CD4^+^ SARS-CoV-2-specific T cells appears to be much higher than in severe disease (112). Strikingly, the pattern of virus-specific CD8^+^ T cell responses to immunodominant epitopes in SARS-CoV-2 is notably different to SARS-CoV, with a large proportion of the total SARS-CoV-2 CD8^+^ T cell responses targeting M, N and ORF3a (53). During acute infection, early T cell responses against ORF7/8 were detected in mild patients, but were virtually absent during convalescence (94). ORF7a has been recently reported to counteract the restriction factor BST2, which tethers newly-produced viruses to the inner plasma membrane impairing viral release (11). These findings taken together could suggest that immune responses against ORF7 are involved in viral control. Also, ORF8 appears to downregulate MHC-I, by targeting it to the lysosomal compartment for degradation, a classic mechanism of viral immune evasion (125). In contrast, responses against structural proteins (S, M, N, and ORF3a) remained unchanged or increased 1 month after infection (94).

Even though the total CD4^+^ T cell count is also reduced in COVID-19 patients, the proportion of CD4^+^ T cells (amongst lymphocytes) appears to remain constant, with numbers of central memory CD4^+^ T-cells found to be markedly increased in severe disease (120). Naïve CD4^+^ T cells, T regs, and effector memory CD4^+^ T cell counts are all lower in acute infection. SARS-CoV-2 infection induces a strong CD4^+^ T cell response that favours Th1 polarisation, but Th2 and Th17 responses have also been detected (106). As virus-specific CD4^+^ T cells are usually key to the production of neutralising antibodies, it is unsurprising to observe a significant correlation between NAb titres and the frequency of virus-specific CD4^+^ T cells (59).

Upregulated expression of inflammatory genes have been highlighted in COVID-19 CD4^+^ T cells, including IL-1ß, FOS, JUN and KLF6 (120). Notably, Th1 cells that express both Interferon gamma (IFN-γ) and GM-CSF were found only in ICU patients with severe disease, with very few found in mild cases and healthy controls, indicating that a subset of T cells induced by SARS-CoV-2 may potentially be pathogenic (126).

COVID-19 T cell responses showed notably higher frequency of S-specific CD4^+^ T cell responses, whilst the majority of CD8^+^ responses were to M, N and ORF proteins (112). SARS-CoV-2 S-reactive CD4^+^ T cells were found in 83% of COVID-19 patients, which targeted both C and N terminal epitopes and expressed markers of recent activation (CD38 and HLA-DR) (59). In a single donor, about 50% of the CD4-specific responses were found to be directed against the spike protein (53).

### Cellular Protection and Cross-Reactivity

T cell responses, and CD8+ responses in particular, may provide durable and robust protection against SARS-CoV-2 infection. The potential for memory T cell responses to clear viruses independent of humoral immunity has been clearly demonstrated for SARS-CoV, as virus-specific memory CD8^+^ T cells were shown to confer protection against lethal SARS-CoV challenge in mice without virus-specific memory CD4^+^ T or B cells (127).

Recall of memory cytotoxic T lymphocyte (CTL) responses against S epitopes could be observed 1 year post-infection with SARS-CoV (128). These epitopes were HLA-A*02:01-restricted (S1203–1211, S978–986 and S1167-1175) and were able to elicit high magnitude IFN-γ T cell responses in recovered patients (129). The S-specific CD8^+^ T cells, targeting S436 or S525 domains, had an effector memory phenotype (CD45RA^+^ CCR7^-^ CD62L^-^) (115, 128) Interestingly, CTL responses could be elicited in a small minority of healthy patients without any history of SARS-CoV infection (129), which suggests that there may be cross-reactive memory T cells naturally present in the T cell repertoire that can elicit recall-like responses following SARS-CoV infection.

Regarding T cell cross-protection for SARS-CoV-2, several groups have demonstrated cross-reactivity for T cell responses, but whether this cross-reaction can provide some level of protection has not yet been addressed (38, 53, 130). Grifoni et al. proposed that immunity generated by HCoV exposure could potentially confer protection against SARS-CoV-2 infection, as they found that 40-60% of unexposed individuals generated CD4^+^ T cell responses against SARS-CoV-2 (53). Similarly, Braun et al. found 34% of healthy individuals to have S-reactive CD4^+^ T cells (130). Interestingly, these SARS-CoV-2 seronegative healthy donors have S-reactive CD4^+^ T cells that exclusively respond to C-terminal epitopes—most likely due to overlapping MHC-II epitopes found on the C terminus of endemic HCoV strains that account for 20% of common colds (130, 131). Le Bert et al. also described SARS-CoV-2-reactive T cells in uninfected individuals, which showed different immunodominance patterns to those found in COVID patients. However, the authors ascribe the epitope recognition to conserved fragments amongst animal CoVs, which show low homology with HCoV (38). In addition, some common immunodominant epitope clusters for S, M, and N proteins were previously identified by Peng et al., but dominant epitopes were shown to have little structural resemblance to common HCoV (112). Another group performed single cell (sc)-RNA sequencing in virus-reactive memory CD8^+^ T cells from convalescent COVID-19 individuals and found similar clusters to pre-pandemic samples when stimulated with SARS-CoV-2 peptides (124).

It will be important to fully elucidate where these T cell epitopes overlap and to determine whether these seronegative individuals show any degree of protection from SARS-CoV-2 infections. Stervbo et al. performed an extensive *in silico* characterisation of the similarity between SARS-CoV-2 antigens and epitopes for the most commonly found pathogens. Several identical epitopes were found for common HCoV HKU1 and OC43 and were predicted to bind HLA-I and -II (132). Despite similar and cross-reacting epitopes, whether there is any contribution to COVID-19 protection from pre-existing cross-reactive T-cell responses is yet to be determined.

Overall, T cell responses appear to be important for good outcomes with COVID-19. SARS-CoV T cell responses have been shown to be able to persist until now, 17 years after the SARS epidemic (38). Evidence points to a lack of a pre-existing immunity offering cross-protection against SARS-CoV-2. However, if T cells from pre-existing immunity to common HCoV are able to cross-react with SARS-CoV-2 there is the possibility that pre-existing immunity might have some effect in mitigating the spread of the pandemic.

## Can Immunopathology in COVID-19 Impair Long-Lasting Protective Immunity?

The current view is that COVID-19 pathophysiology is exacerbated by immunological dysregulation. In severe cases, lymphopenia and dysregulated inflammatory cytokine production account for an excessive immune response leading to a cytokine storm, which in turn can provoke tissue damage, ARDS and multi-organ failure (35, 132). Single-cell RNA-seq from PBMCs from mild and severe COVID patients found a hyper-inflammatory signature in all cell types linked to TNF/IL-1ß-driven inflammation, which co-existed with a strong type I IFN-driven inflammatory response in severe patients (134). In a longitudinal study of patients admitted to UK hospitals, the degree of hyper-inflammation on admission [defined as COV-HI (hyperinflammatory), assessed using plasma C-reactive protein >150 mg/ml or doubling within 24 h and Ferritin >1500 mcg/ml] strongly predicted poor outcomes (135).

Does this hyperinflammatory response impair the formation of a memory compartment and subsequent protective immunity? There is no conclusive evidence yet to suggest an impaired memory compartment in patients with severe COVID-19 compared to those with mild disease. However, the lack of GC formation in severe COVID-19 patients may suggest that despite the high antibody titres linked with severe disease, the humoral response for these patients may be short-lived (80). The significant T cell lymphopaenia of severe cases may also influence whether a successful antibody- and T cell-mediated response can be mounted during this phase of inflammatory drive.

Also, T cell memory formation is known to be impaired in the context of chronic inflammation (136). Whether this holds true in the context of the cytokine storm in response to SARS-CoV-2 infection, and whether this leads to some level of impaired long-lasting T cell immunity is still unknown. There is an urgent need to understand whether the immune response generated after severe COVID-19 with excessive immune activation (COV-HI) differs from that which develops in mild cases. If a primary SARS-CoV-2 immune response cannot fully develop in critically ill patients, the formation of memory immune cells may also fail. Therefore, the differences in the adaptive immune response generated in mild compared to very sick patients, such as antibody titres and epitope targets of antibodies and T-cells, and the adequate formation of the memory compartment are likely to influence the level of protection against SARS-CoV-2 reinfection.

### Variability of The Immune Landscape

Prior immune status has been known to play a role in many disease outcomes. Previous infections or co-morbidities can additionally modify the immune landscape. Co-infection with persistent viruses such as human cytomegalovirus (CMV) are known to facilitate disease progression in AIDS by driving immune activation (137), whilst co-morbidities such as obesity, associated with severe COVID-19, lead to a state of chronic inflammation and impaired immune function (138). The immune status generated by co-infections and pre-morbid conditions may correlate with distinct COVID-19 outcomes and warrants further investigation.

It is also possible that genetic differences can influence different immunophenotypes with distinct disease outcomes following SARS-CoV-2 infection. Two recent publications show a clear relationship between life-threatening COVID-19 and defects in the Type I IFN response, either from inborn errors in the IFN signalling pathway (36) or through the acquisition of auto-antibodies against IFN (37). Different immunotypes have been proposed for COVID-19 patients based on integration of B and T cell responses. Differential activation for either CD8^+^ and CD4^+^ T cells was observed in subgroups of patients. The same study segregates COVID-19 patients into two distinct patterns for activated B cell responses and identifies a third group with little evidence of an active humoral response (119).

Recent genome-wide association studies (GWAS) and large-scale immunophenotyping studies have compared immunological and genetic profiles between patient outcomes in sepsis (139). Similar functional genomic studies are warranted for COVID-19 to elucidate the patient-to-patient variability that will help us understand if genetics play a role in why a few individuals undergo severe immunopathological responses whilst most do not (140). To date, the largest GWAS of COVID-19 genetics published has identified a six-gene cluster on chromosome 3p21.31 which shows a significant association with severe disease (141). The risk genotype was found to be present at significantly higher frequency in patients who required mechanical ventilation. The locus contains genes implicated in immune regulation, including SLC6A20 which interacts with ACE2 and CXCR6—a receptor known to be expressed in lung-resident T cells and plays a role in the regulation of the inflammatory response to airway infections (142, 143). In addition, genetic variability in Human Leucocyte Antigen (HLA) class I has been shown to affect susceptibility to severe disease in COVID-19, with patients with the HLA-B*46:01 allele being particularly vulnerable (144). Further genetic studies will shed more light on why patient outcomes for Covid-19 can be so dramatically different.

Addressing whether the differences in the host immune response to SARS-CoV-2 is owed to the individual’s previous immunological history, or the result of genetic predisposition, and whether this affects the generation of memory responses will be an important consideration in the development of vaccine strategies.

## Vaccine-Induced Immunity

As of 29^th^ October 2020, a total of 45 candidate vaccines against COVID-19 are in clinical trials worldwide, with a further 156 other candidates in pre-clinical evaluation (145). Of those, six vaccines have been approved for early or limited use: one by CanSino, Sputnik V by the Gamaleya Research Institute, EpiVacCorona by the Vector Institute, CoronaVac by Sinovac Biotech, and two by Sinopharm together with the Wuhan Institute and the Beijing Institute of Biological Products. Among the approved vaccines, three contain inactivated virus, two use non-replicating adenoviral vectors and one is protein-based. A total of 10 vaccines are in Phase III clinical trials—including the aforementioned approved vaccines and others which are the first of their kind to enter Phase III—like the mRNA based vaccine by Moderna and the ChAdOx1-vectored vaccine from Oxford/AstraZeneca (145).

The S and the N proteins have proved to be powerful immunogens for vaccine development, as they elicit both humoral and cell-mediated responses, for both SARS-CoV and SARS-CoV-2, as well as and MERS-CoV (27, 105, 146). The general consensus from human studies concludes that the S protein from SARS-CoV is capable of inducing neutralising antibodies as well as CD4^+^ and CD8^+^ T cell responses, whilst the N protein mostly elicits T cell responses (146, 147).

Following the first SARS epidemic, S protein-expressing vaccines against SARS-CoV were shown to elicit protective immunity mediated by neutralising antibody production (28, 148, 149). S protein-based DNA vaccines induced high titres of potent neutralising antibodies that were able to inhibit viral entry but lacked cell-mediated protective efficacy against SARS-CoV (28). However, later animal studies showed that in mice, S-specific memory CD8^+^ T cells could also elicit protective immunity, even in the absence of SARS-CoV-specific memory CD4^+^ T cells or B cells, after a challenge with a lethal dose of the virus (127). Subsequent trials of N protein encoding DNA vaccines and synthetic N peptide vaccines elicited potent CD8^+^ T cell responses that conferred protection in SARS-CoV challenge models (150–152). In early preclinical studies of SARS-CoV vaccines conducted in mice, immunisation with N protein-based DNA vaccines have demonstrated induction of potent CTL responses (150). This preclinical evidence, combined with the observations of high neutralising antibody titres in recovered patients, provided good reason to believe that anti-S protein antibodies and CTL responses against N proteins were likely to be required to provide immunity against SARS-CoV. However, the disappearance of SARS-CoV infection prevented efficacy testing of these vaccines.

In the race to develop a SARS-CoV-2 vaccine, the first results have shown that an S-based DNA vaccine in a prime-boost regime in macaques induces NAb titres similar to those found in recovered humans and macaques (32). These antibodies showed better neutralising capacity following vaccination using the full prefusion-stabilised S protein. When challenged with SARS-CoV-2, vaccine-elicited NAbs appeared to be the major immune correlate of protection, together with some innate effector functions directly related to antibody efficacy (39). The *ChAdOx1 nCoV-19* vaccine, another ongoing vaccine trial based on a ChAd (Chimpanzee Adenovirus) viral vector expressing the S protein, demonstrated some efficacy in preventing pulmonary pathology following SARS-CoV-2 challenge in non-human primates (98). All immunised macaques (6/6) were protected from developing pneumonia, which was seen in the control group (2/3). The immunised group had significant reductions in viral load in BAL fluid, but no differences were seen in viral load in nasal swabs (NS) compared to controls. Immunisation appears to reduce the severity of pathogenesis without any significant reduction in viral shedding. The authors found no sign of ADE and ascribe the persistence of virus in nasal swabs to the high titres of virus used for the challenge (98). These results are encouraging and could suggest that these first vaccines may provide protection against severe illness, even if they fall short of providing sterile immunity.

In humans, two vaccinations of the mRNA-1273 vaccine, known as the Moderna vaccine and based on a lipid nanoparticle that encapsulates RNA encoding S glycoprotein, have so far proven to elicit antibody and CD4^+^ T-cell responses but low CD8^+^ T-cell responses (153). Published Phase I/II data from *ChAdOx1 nCoV-19* demonstrates the immunogenicity of the S protein, eliciting both neutralising antibodies and virus-specific effector T cell responses, with no major adverse effects (154). Although neutralising antibodies were induced by a single dose, a prime-boost regimen was shown to induce significantly higher titres of neutralising antibodies—a magnitude that, in a previous macaque study, appeared to be adequate for conferring protection against reinfection (40, 154). At least 10 of the vaccines now in Phase III trials report the induction of NAb titres in response to vaccination (155–157). Though limited data have been reported on cellular immune responses, all 10 appear to generate a detectable virus-specific T cell response in IFN-γ assays, although these vary in magnitude. At this point, it is not certain if vaccine-induced immune responses are durable when compared with natural infection. But results suggest that the induction of a robust virus-specific T cell responses with Th1 type polarisation is certainly possible.

From the published results from phase I/II trials across a range of vaccine subtypes, we find that adjuvanted protein based vaccines are the most immunogenic, followed by mRNA vaccines, followed by ChAdOx1 and inactivated vaccines, with AdV5-based vaccines being the least immunogenic in terms of NAb titres, although it is difficult to directly compare these studies due to differences in the assays being used (155). The AdV5-based vaccines (CanSino) were shown to be reactogenic, especially at higher doses and in older patients who are likely to have pre-existing immunity to AdV5 (158). Certain phase III candidates including the inactivated and mRNA vaccines (from Sinovac and Pfizer respectively) have already shown significantly lower immunogenicity in the older population, and may require booster regimens or higher doses in order to generate a durable protective efficacy—this is expected, as it has also been the case for the influenza vaccine (159–161).

Although these studies note the generation of T cell responses against the S protein, the extent to which protection is owed to the presence of S-specific CD8^+^ T cells is not addressed. The mRNA-1273 (Moderna) vaccine, for example, showed good CD4+ responses and detectable, but low, CD8+ T cell responses against the S protein (153). It was recently shown that patients who recovered from mild disease possess a higher frequency of CD8^+^ T cells specific for M or N proteins rather than to the S protein when compared to severe cases (112). The same study points out that though the S protein generates a substantial CD8^+^ T cell response, it does not appear to be immunodominant (112). This potentially has major implications for candidate vaccines using solely the S antigen, as these might elicit a narrower CD8^+^ T cell response than natural SARS-CoV-2 infection. An effective CD8^+^ T cell-inducing vaccine may therefore require additional antigens beyond the spike protein. Emerging studies are identifying epitopes across the SARS-CoV-2 genome predicted to activate both CD4^+^ and CD8^+^ responses that may induce long-lasting immunity (125). The combination of different epitopes may help build a robust memory response to provide protection through a powerful and effective vaccine.

Additionally, the majority of the current vaccines in clinical trials are administered through the skin or muscle. This route of administration may fail to generate a protective immune response in the upper respiratory tract that would otherwise be generated in natural infection (98). Hassan et al. demonstrated that a single-dose of intranasal administration of a ChAd-vectored S vaccine induced a local immune response that was sufficient to provide sterilising immunity in mice (163). There is reason to believe that local immunisation with a vaccine that can generate an IgA mucosal antibody response in the upper respiratory tract may provide a robust strategy for effective early clearance, as shown in successful trials of measles and BCG vaccines administered through the aerosol route (164, 165).

## Discussion

Emerging evidence from recent SARS-CoV-2 reports, combined with literature from nearly two decades of SARS-CoV research, provide good reason to believe that it should be possible to generate protective immunity against SARS-CoV-2 in humans, either following natural infection or with a vaccine. Long-lasting protective immunity is likely to require both a sufficient titre of circulating NAbs and a strong T cell response, consisting of virus-specific central memory CD4^+^ and effector memory CD8^+^ T cells. Whether natural infection will induce a humoral and cell-mediated immune response that provides long-lasting protection against reinfection, and how this compares with the immune response generated by vaccination, and whether the inflammatory response will impair the proper formation of a memory compartment currently remains unknown.

The development of a safe SARS-CoV-2 vaccine is made more complex because of evidence of an immunopathological response underpinning disease severity. Antibody-dependent enhancement, along with direct tissue damage to alveolar cells, have been noted amongst studies conducted in SARS-CoV, and therefore warrants caution in future SARS-CoV-2 vaccine studies. Vaccine candidates using full-length S protein sequences may be counterproductive for certain individuals who may possess a predisposition for generating an immunopathological response to this antigen: the biological basis of adverse responses to S proteins needs to be elucidated.

The recent studies in macaques seem to suggest that counterproductive responses are not observed with the SARS-CoV-2 S protein, and induction of high titres of anti-RBD neutralising antibodies are likely to confer protection with minimal risk of causing an immunopathogenic response, provide a promising avenue for vaccine development. One caveat for vaccines that solely utilise the S protein is that this may well lead to a narrower cellular immune response and reduce the spectrum of antibodies induced – potentially reducing protective efficacy. However, the data from the vaccines currently in clinical trials appear promising, with evidence of robust neutralising antibody titres and various degrees of virus-specific T cell responses against the S protein. Whether or not strong immune responses to the S protein alone will provide adequate protection is still unclear, but the limited evidence from the animal challenge studies previously discussed seem to indicate that this may be sufficient to protect against severe disease. More recently, trials deploying intranasal immunisation using the S protein have been shown to elicit a broad spectrum of antibodies and T cell responses, including local mucosal responses, which may be a promising avenue for improving vaccine efficacy (163).

We are beginning to decipher the differences in immunological profiles between individuals who suffer from severe illness and those with asymptomatic or mild disease. In a similar manner to autoimmune diseases, this dysregulated response may occur only in a particular group of the general population that possess a predisposition to severe disease, based perhaps on initial infectious dose, viral and host genetics, and the individual profile of previous antigenic exposures. For this reason, large-scale immunophenotyping of COVID-19 patients has an important place on the global research roadmap for vaccine development.

Importantly, based on the preclinical studies conducted in SARS-CoV, there still remains a small possibility of generating an immunopathological response through vaccination that causes disease enhancement (86). So far, animal studies for vaccine candidates for SARS-CoV-2 have shown no evidence of immune-enhanced pathology or a Th2 biased response (39, 98), and all 10 candidates in phase III clinical trials have, to date, shown no adverse events directly linked to the vaccine immunisation that warrants concern about disease enhancement (145)

Overall, the evidence from observational studies in both SARS-CoV and SARS-CoV-2 infection, along with the promising data from clinical trials across 42 vaccine candidates, provide sufficient reason to speculate that a vaccine for SARS-CoV-2 will be safe and could provide lasting protective immunity that even if not lifelong might persist for years. Future studies, in due course, are expected to provide conclusive evidence about whether NAbs and/or virus-specific T cell responses will provide protection against infection, transmission and severe disease. Understanding the magnitude and characteristics of virus-specific cellular responses or titres of antibody responses required to provide protection will guide both vaccine design and public health policies to limit spread.

## Author Contributions

All authors contributed to the article and approved the submitted version.

## Conflict of Interest

The authors declare that the research was conducted in the absence of any commercial or financial relationships that could be construed as a potential conflict of interest.
